# Gonadal Y-chromosome mosaicism with 45, X Turner syndrome complicated with bilateral HCG-secreting gonadoblastoma

**DOI:** 10.3389/fped.2022.1042427

**Published:** 2022-11-22

**Authors:** Rujiang Zheng, Qiuli Chen, Huamei Ma, Juncheng Liu, Huadong Chen, Jianbo Liang, Hongshan Chen, Jun Zhang, Yanhong Li, Song Guo, Bing Wang, Minlian Du

**Affiliations:** ^1^ Department of Pediatrics, The First Affiliated Hospital, Sun Yat-Sen University, Guangzhou, China; ^2^ Department of Pediatric Surgery, The First Affiliated Hospital, Sun Yat-Sen University, Guangzhou, China; ^3^ Department of Medical Laboratory, The First Affiliated Hospital, Sun Yat-Sen University, Guangzhou, China

**Keywords:** Turner syndrome, gonadoblastoma, Y chromosome, human chorionic gonadotropin, precocious puberty

## Abstract

We report a rare case of bilateral HCG-secreting gonadoblastomas (Gb) in a 5.25-year-old girl of 45, X Turner syndrome (TS) with gonadal Y chromosome mosaicism. The clinical data were summarized, and the literatures were reviewed. The patient had enlarged breasts for 2 years and 3 months, with elevated β-HCG of blood found for 8 months. The level of β-HCG of cerebrospinal fluid, cranial MRI, chest and abdominal CT, and pelvic MRI were normal. After surgical gonad exploration, biopsy and excision, gonad venous blood hormone examination and SRY gene detection of gonad tissue, the diagnosis was confirmed as HCG-secreting Gb (bilateral) and TS (45, X) with gonad Y chromosome mosaicism. The patient received 4 courses of chemotherapy, and regular outpatient follow-up. At 9 months after gonadectomy, there was no clinical, laboratory, or radiological evidence of recurrence. We reported a nonclassical case of 45, X Turner syndrome (TS) with gonadal Y chromosome mosaicism, who presented with breast development as the first manifestation and then virilization due to bilateral HCG-secreting gonadoblastomas. Detection of serum β-HCG and AFP is requisite for the diagnosis of precocious puberty, karyotyping is important for virilizing phenotypic female, and virilization in Turner syndrome implies the existence of Y chromosome(substance) (peripheral blood or tissue mosaicism) and the occurrence of gonadal tumors.

## Background

Turner syndrome (TS) is one of the most common sex chromosome anomalies, with a prevalence of 1:2,000–1:4,000 live-born girls. It's characterized by a complete or partial loss of one X chromosome (1) or structural abnormalities of the X chromosome (2), and its clinical manifestations include short stature, gonadal dysplasia, and specific physical features (e.g., webbed neck, pelvis, cubitus valgus) among others (1, 2). Patients with TS may have an abnormal Y chromosome or mosaicism. Gonadoblastoma (Gb) is common among TS patients with Y-chromosome components, although HCG-secreting Gb is rare. We report a case of HCG secreting Gb in a TS (45, X) patient with Y chromosome mosaicism who complained of breast development and exhibited virilizing features. We also performed a review of literature to inform on the diagnosis and treatment of TS complicated with HCG-secreting gonadal germ cell tumors (GCTs).

## Patient Presentation

A 5-year 3-month-old girl was admitted to our hospital with a history of enlarged breasts for 2 years and 3 months, as well as elevated β-HCG for 8 months. When the patient's chronological age (CA) was 3 years, she was found to have enlarged breasts, prompting her to visit a local hospital for examination. At that time, the physical examination for this patient revealed: height (Ht) 93 cm (−1SD), weight (Wt) 14.05 kg (0SD), Tanner stage: B3 (diameter of the mammary gland nodes: 2.5 cm), and a grade 2–3/6 systolic murmur audible on split second heart sound. Blood tests revealed: follicle-stimulating hormone (FSH) 0.83 IU/L, luteinizing hormone (LH) 0.04 IU/L, estradiol (*E*_2_) <10 ng/L, testosterone (T) 0.71 μg/L, and prolactin (PRL) 12.31 μg/L. Bone age (BA) was 3 years old. Gynecological B ultrasound: uterus 16 mm × 13 mm × 10 mm, left ovary (LO) 14 mm × 10 mm × 6 mm, and right ovary (RO) 15 mm × 11 mm × 6 mm. Echocardiography: partial pulmonary vein ectopic drainage and atrial septal defect (secondary hole type). She underwent “Pulmonary venous malformation drainage correction and Secondary hole atrial septal defect repair” in another hospital when her CA was 3 years and 4 months. She recovered well after surgery, but her breasts continued to rapidly grow and enlarge.

**Figure 1:**
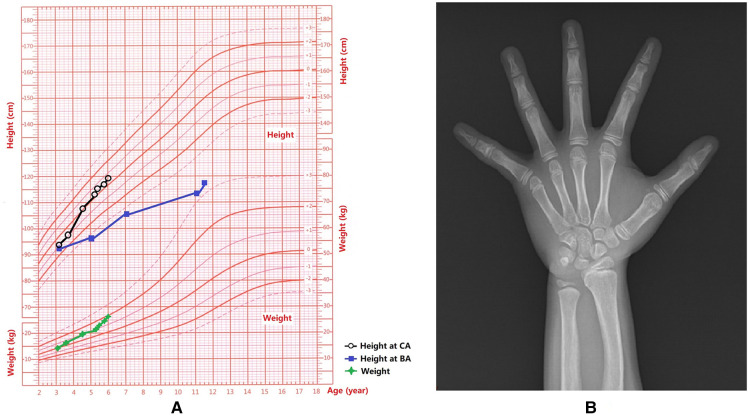
Growth and development data of this patient. (**A**) Weight and height for each age stage; Chronological age, CA; Bone age, BA. (**B**) The *x*-ray showed that the BA was 10.5 years old and there was a slight Madelung deformity when the patient was 5 years and 3 months old.

At the age of 3 years and 7 months, she visited the local hospital again. Physical examination revealed that her height was 97 cm (−0.8SD). BA: about 5 years old. Gonadotropin-releasing hormone analogues (GnRHa) stimulation test (60 min): FSH_0–60_ _min_ 1.46–10.30 IU/L, LH_0–60_ _min_ <0.3–3.46 IU/L. Blood test: *E*_2_ 15 ng/L, T 0.45 μg/L. No definite pathogenic gene mutation was identified in the whole exome sequencing test. 45, X, was the peripheral blood chromosome. Re-examination of the patient at the CA of 4 years and 6 months revealed: blood β-HCG 24.94 IU/L, alpha-fetoprotein (AFP) 0.77 μg/L, basal FSH 0.46 IU/L, LH 0.15 IU/L, and peak LH/FSH was 0.25 after GnRHa stimulation test. Pituitary MR scan as well as chest, adrenal gland, and pelvic CT scans did not reveal any abnormalities. In the higher-level hospital, further diagnosis and treatments were recommended. Due to rapid breast development, the patient experienced accelerated height and weight gain (Figure 1A), and she was energetic without any discomfort. She was admitted to our hospital at the age of 5 years and 3 months. Physical examination on admission revealed: Blood pleasure (BP), 103/71 mmHg; Ht, 113.7 cm (+0.1 SD, growth velocity =10 cm/year); Wt, 21.8 kg (+1 SD) [Figure 1A (3)] and BMI, 16.86 kg/m^2^ (P90th). Her heart rate was 100 beats/min, the rhythm was regular, and a grade 2/6 systolic murmur was audible in the second intercostal space on the right sternum. Tanner staging: A1, B3 (diameter of the mammary gland nodes: 9.0 cm), PH3, female vulva, and clitoral hypertrophy (2.5 cm × 1.0 cm).

Laboratory examination revealed: urine RBC was 0–3/HP; blood routine, biochemistry, liver enzymes, liver function, blood lipids, and thyroid function were all normal; lactate dehydrogenase (LDH) 244 IU/L (<250.00 IU/L); insulin-like-growth-factor-1 (IGF-1) 230.00 μg/L (143.70–319.10 μg/L); 8AM cortisol 8.90 ug/dl (5.00–19.40 ug/dl); adreno-cortico-tropic-hormone (ACTH) 8.61 pmol/L(4.50–18.00 pmol/L); 17α-hydroxyprogesterone 0.56–1.27 μg/L (<2.00 μg/L); androstenedione <1.05–1.66 nmol/L; dehydroepiandrosterone (DHEAs) 0.25–0.28 umol/L; Basal FSH 0.12–0.16 IU/L; LH 0.02–0.03 IU/L; PRL 11.72 μg/L (<30.00 μg/L); *E*_2_ 28.00–30.00 ng/L (<10.00 ng/L); β-HCG 32.33–46.02 IU/L (<1.20 IU/L); AFP 0.94–0.90 μg/L(<20.00 μg/L); T 1.96–2.53 μg/L (<0.27 μg/L); progesterone (P) 0.10 μg/L; anti-Müllerian hormone (AMH) 0.57 μg/L (0.05–7.02 μg/L) (4). Inhibin B < 10 ng/L (0.00–50.23 ng/L) (4); Cerebrospinal fluid (CSF) β-HCG < 1.20 IU/L; and CSF-AFP 0.01 μg/L (at the same time peripheral blood 0.85 μg/L).

BA was 10.5 years old (Figure 1B). Echocardiogram results: following pulmonary venous drainage and repair of the atrial septal defect, a small shunt at the atrial level was 1.8 mm, the left atrium was slightly enlarged. Breast B-ultrasound revealed double breast cysts. Gynecological ultrasound revealed a linear endometrium, left ovary measuring 12 mm × 9 mm with 1 antral follicle (4 mm × 5 mm), and right ovary measuring 12 mm × 8 mm with no antral follicles ≥4 mm in diameter. Chest and abdominal CT revealed bilateral breast development, bilateral renal malrotation, and bilateral extrarenal renal pelvis. Thyroid and abdominal ultrasounds, spine *x*-ray, MR of the pelvic cavity, and the hearing test did not reveal any abnormalities.

Peripheral blood SRY gene (Polymerase chain reaction, PCR) was negative. Abnormal peripheral blood sex chromosome number detection (FISH, CEPX/Y probe) showed that there were 494/500 cells exhibited 1 X centromere, 6/500 cells exhibited 2 X centromeres, and there was no Y centromere signal.

## Diagnosis

Based on the above findings, the patient was 45, X TS, with masculine characteristics, and peripheral blood T levels progressively increased. The patient was postulated to have a Y chromosomal material. The SRY gene was negative and there was an abnormal number of blood sex chromosomes (FISH, CEPX/Y probe), indicating the absence of Y chromosomal material in peripheral blood. Therefore, we suspected that the patient had a gonad tumor harboring Y-chromosomal material and secreting hormones such as *E*_2_, T, etc., and there were indications for gonadal examination.

This patient had elevated β-HCG in peripheral blood, negative imaging examination and nomal β-HCG levels in the CSF which did not support intracranial or extragonadal tumors. These findings suggest that gonads are the primary sites for HCG secretion, and should therefore be studied further. According to monism, HCG-secreting Gb is conceivable. However, HCG-secreting Gb is extremely rare, with only 4 cases reported internationally; therefore, gonad exploration and pathology can explain for the above concerns.

## Treatment

### The first operation

**Figure 2:**
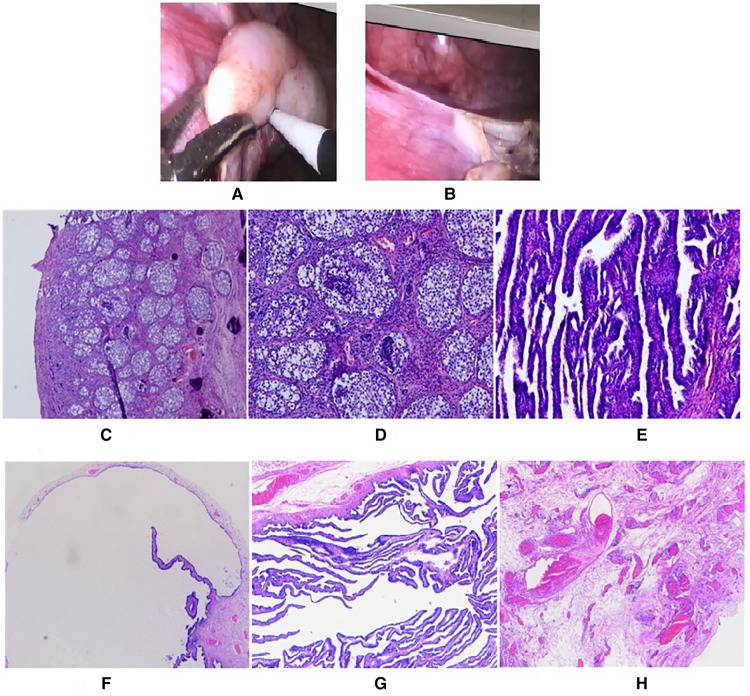
Gonadal morphology and pathological biopsy in this patient. (**A**) The left gonad was of acceptable size, but was nodular. (**B**) The right gonad was cord-shaped, with a few suspicious follicular structures. (**C–H**) Pathological biopsy and immunohistochemistry.

With the consent of parents, surgical exploration was performed 28 days after admission (CA: 5 years and 4 months old). Laparoscopic examination revealed that the left gonad was of acceptable size (Figure 2A); however, it was nodular. The right gonad was cord-shaped (Figure 2B), with a few suspicious follicular structures. Given the preoperative hormone levels and intraoperative diagnosis of bilateral gonadal dysplasia, the left gonad was thought to be a tumor. However, the parents only agreed to the left gonadectomy and the right gonad biopsy, but did not agree to the right gonadectomy. During the operation, a few blood samples were drawn from bilateral gonadal veins and peripheral veins for hormone level assessment. Bilateral gonadal veins had significantly elevated levels of β-HCG, *E*_2_, and T levels (Table 1). The entire left gonad was removed, and a small sample of tissue, left and right, up and down of the right gonad was collected for pathological examination. Three tissues from the left gonad and one from the right gonad were collected for SRY gene detection (PCR), all of which were positive (Table 2).

**Table 1:** The hormone level from bilateral gonadal veins and peripheral vein.

	β-HCG (IU/L)	AFP (μg/L)	*E*_2_ (ng/L)	T (μg/L)
Left gonadal vein	52.72	0.40	758.00	16.40
Right gonadal vein	43.83	0.70	90.00	4.73
Peripheral blood	40.43	0.65	24.00	0.87

β-HCG, β-human chorionic gonadotropin; AFP, alpha-fetoprotein; *E*_2_, estradiol; T, testosterone.

**Table 2:** SRY gene PCR detection.

Peripheral blood	Left gonadal	Right gonadal
(–)	Outer (+)	Outer upper edge (+)
	Middle (+)	
	Inner (+)	

Pathological biopsy and immunohistochemistry suggested bilateral Gb (Figures 2C–H). In all the 4 specimens of the left gonad and the inner and lower border tissues of the 4 specimens of the right gonad, nested tumor cells could be seen in the tissue. Abundant, with large nuclei, and some cells were small in size; cystic wall-like structures were seen in focal areas, and the surface was covered with monolayer or stratified squamous epithelium. Under microscope, there were scattered light red-stained basement membrane-like substances and numerous calcifications in tumor cell nests. Tumor cell-related SALL4, OCT3/4, PLAP, CD117, and D2–40 were all positive. AFP was focally positive, with individual positive HPL cells; Inhibinα, CR, and HCG were all positive; SF-1 was partially positive; A few WT1 cells were positive; CD99 was weakly positive; CK, CD30, Glypican-3, Hepatocyte, AR, Melan-A were all negative, and about 30% of Ki-67 were positive.

### The second operation

After being informed of the pathological results and waiting for another five working days, the family finally consented to a re-surge of the right gonad. Laparoscopic and right gonadectomy was performed on the 26th day after the first operation. Pathologyical results revealed mesosalpinx cyst and a large amount of tubal-like tissue, in addition to the presence of scattered inflammatory cell infiltration and focal multinucleation on the local peripheral serosal surface. Giant cells aggregated, consistent with postoperative inflammatory changes, but there was no tumor or malignant features.

### Chemotherapy

After operation, the patient was referred to the Children's Oncology Department of Sun Yat-sen University Affiliated Cancer Hospital for consultation.Since the tumor secreted HCG, four courses of chemotherapy (etoposide, carboplatin, etoposide, carboplatin, etc.) were administered 3 months after the first operation.

## Outcomes

After surgery, the rapid increase in the patient's height slowed down (Figure 1A), her breasts shrank, her glandular nodes softened, her clitoris shrank and softened, her pubic hair completely disappeared half a year later, and bone age progression slowed down (Figure 1A).

Twenty hours after the first operation, peripheral blood *E*_2_ and T levels were significantly decreased to below the limit of detection. After surgery, there was a significant decrease in β-HCG levels, which did not return to normal at 68 h (12.9% of the intraoperative percentage), and were below the detection limit 8 days after surgery. At last follow-up (258 days following surgery), peripheral blood *E*_2_, T, and β-HCG levels were all below the detection range. There were no tumor recurrence indications (Table 3).

**Table 3:** Changes of hormone levels after operation.

	FSH (IU/L)	LH (IU/L)	*E*_2_ (ng/L)	T (μg/L)	β-HCG (IU/L)	AFP (μg/L)
Preoperation						
−42d	0.12	0.02	28.00	1.96	32.33	0.94
−27d	0.16	0.03	30.00	2.53	51.27	1.42
−24d					46.02	0.90
−23d					50.53	0.85
−6d					37.01	0.75
0d	**First operation (Left gonadal resection, right gonadal biopsy)**					
Intraoperative			24.00	0.87	40.43	0.65
Postoperative^a^						
20h	0.28	0.04	<10.00	<0.13	17.71	0.66
68h					5.23	0.65
8d					0.30	<1.80
25d	2.16	0.21	<10.00	<0.13	<1.20	0.70
26d	**Second operation (Right gonadal resection)**					
88d					0.07	0.94
91d		1.41	20.20	0.17	<1.20	1.05
	**Four courses of chemotherapy**					
113d					<1.20	1.05
133d					<1.20	
159d					<1.20	
179d	9.04	0.22	<10.00	<0.13	<1.20	1.12
258d	4.31	0.14	<10.00	<0.13	<1.20	2.00

d, day; h, hour; FSH, follicle-stimulating hormone; LH, luteinizing hormone; *E*_2_, estradiol; T, testosterone; β-HCG, β-human chorionic gonadotropin; AFP, α-fetoprotein.

^a^ Postoperation refers to the first postoperation.

## Literature review and discussion

A review of the literature is presented, with a summary of 4 articles and our case of Gb secreting HCG (Table 4). We used the search terms “gonadoblastoma” and “HCG” on PubMed to get series of articles. There are few reports on secretion of HCG by Gb, and only 4 cases have been reported. In 1976, Ishida (5) reported that a 17-year-old Caucasian girl had a karyotype of 45, X/46, X, r(Y), and Gb as a result of secondary amenorrhea and masculinization characteristics. The levels of *E*_2_, T, and β-HCG in tumor tissue effluent and peripheral blood were elevated; however, there were no trophoblast cells in the tumor tissue. They hypothesized that histologically undifferentiated germ cells are capable of secreting HCG. In 1990, Fukamatsu (6) reported a case of bilateral Gb in a phenotypic female with 45, X/46, XY gonadal dysgenesis, secondary amenorrhea for 2 years, and mild virilization. The levels of β-HCG were preoperatively elevated (5.0 IU/L), but decreased to 3.0 and 1.0 IU/L at 7 and 14 days after operation. However, immunohistochemical analysis of HCG was negative. In 1998, Lange (7) reported a case of 46 XY gonadal dysgenesis (Swyer-syndrome) with bilateral androgen producing Gb in streak gonads in a 15-year-old patient. Preoperative androgen and β-HCG levels were high and decreased to normal after surgery. In 1999, Schanne (8) reported the case of a patient with pure gonadal dysgenesis, XY karyotype, and elevated peripheral serum β-HCG (42.4 IU/L) after a positive pregnancy test. However, However, whole-body imaging examination did not reveal any other HCG secretion lesions, and they returned to normal after operation. Immunohistochemistry of the pathological tissue revealed HCG in a few mononuclear cells. Our case represents the fifth case of HCG-secreting Gb to be reported globally. Additionally, this case is the first that presented in early childhood and all the others presented in puberty (Table 4), in line with the general accepted theory that puberty of gonadotrophins/central initiated growth and de-differentiation of the gonadal dysgenesis. Our patient presented with breast development, masculine features such as firm muscles, pubic hair development, clitoral hypertrophy, and elevated T and β-HCG in peripheral blood. The immunohistochemistry performed on the pathological tissue revealed that certain cells were positive for HCG. In addition, whole-body imaging examination was performed to identify any other HCG-secreting lesions.

**Table 4:** Comparison of general data of 5 cases of Gb secreting HCG.

Reporter/s	Age (years)	Sex	Karyotype	Tanner stage	Lesion site/Pathology	IHC-HCG	Peripheral blood HCG levels	HCG levels of gonadal venous or tissue	Postoperative HCG levels
Ishida (5)	17	Female	45, X/46, X-ring-y	PH4, B4-5	Left gonad/ Gb	–	74.40 IU/L	156.00 IU/L	Normal
Fukamatsu (6)	17	Female	45, X/46, XY	PH3, B3	Bilateral gonad/ Gb	(−)	5.00 IU/L	–	Normal
Lange (7)	15	Female	46, XY	PH4, B4	Bilateral gonad/ Gb	–	40,456.00 IU/L	–	Normal
Schanne (8)	16	Female	46 XY	PH3, B4	Left gonad/ Gb	(+)	42.40 IU/L	–	Normal
This case	5	Female	45, X	PH3, B3	Bilateral gonad/ Gb	(+)	37.01–51.27 IU/L	43.83–52.72 IU/L	Normal

HCG, human chorionic gonadotropin.

Individuals with 45, X are the only survivors of the chromosomal monosomy syndrome; they are predominantly female, with a few male phenotypes; the 45, X karyotype accounts for 40%–50%, while mosaicism accounts for 20%–50% (1, 2). The surviving 45, X individuals exhibit a certain degree of mosaicism, implying that the karyotype of some cell lines is not 45, X (1). In our case, her karyotype was 45, X, peripheral blood SRY (-) and FISH (CEPY /CEPX probe) did not reveal any corresponding Y-chromosomal material. This suggested that the patient's gonads had T- and HCG-secreting tumors (such as Gb), in addition to the presence of Y-chromosomal material (gonadal tissue mosaicism). The final diagnosis of 45, X TS, and HCG-secreting Gb with Y chromosome mosaicism was confirmed after laparoscopic exploration, examination of gonadal venous blood concentration, gonadal excision/biopsy, SRY gene, and immunohistochemical detection of bilateral gonadal tissue. These findings suggest that patients with TS have an increased risk of developing central nervous system tumors, meningiomas, bladder and urethral tumors, as well as melanoma (9), but a low risk of breast cancer development (10). However, these patients do not have an increased risk of tumor development (10, 11). Since about 10% of TS patients have a Y-chromosomal material component, the Y component significantly increases the risk of Gb by 5%–30% (1, 2), and it may also increase the risk of androgen-secreting non-neoplastic lesions.

Gb, which was first described by Scully in 1953, is a mixture of seminoma-like large germ cells and small sex cord-stromal cells such as immature Sertoli cells and granulosa cells. It is a premalignant state (benign tumor) along with germ cell neoplasia *in situ* (GCNIS) of the testis (12, 13). About 50%–60% of Gb patients progress to dysgerminoma, while 10% progress to non-seminoma GCT, which is listed in the 2020 WHO classification of ovarian tumors as a germ-cell-sex cord-stromal tumor (14). Typically, Gb occurs in cord-like or dysplastic gonads, which are found in patients with disorders of sexual development (DSD).

Clinical manifestations of Gb are variable and may include asymptomatic, female phenotype without virilization, female phenotype with virilization, and pure virilization (12). Gonadal germ cell tumors in TS patients often exhibit virilization, although breast development is an extremely rare symptom of these tumors. In this case, the patient presented with significant breast development, cystic hyperplasia, and masculine features.

It is possible for peripheral blood sex hormone levels to be normal in Gb patients, and it is also possible for T, *E*_2_, P, DHEAs, and androstenedione levels to be elevated. Elevated PRL levels have been reported (15). Based on peripheral blood and gonadal venous blood hormone concentrations of this patient, we hypothesized that her gonads had tumor tissues that secreted *E*_2_, T, and β-HCG. Studies should determine whether elevated testosterone levels in these patients are secreted by Gb, or whether Gb secreted by HCG stimulates the maturation of sex cord stromal cells. We speculate that the high levels of *E*_2_ and T secreted by long-term gonadal tumors has been secondary to the partial activation of hypothalamic pituitary gonadal axis function, ultimately enabling breast development in our patient.

HCG is a glycoprotein hormone that is secreted by placental syncytiotrophoblast cells. Its primary function includes the maintenance of corpus luteum, promotion of fetal development, and immunosuppression. Under normal circumstances, elevated HCG levels may be considered a pregnancy, ectopic pregnancy, or old ectopic pregnancy. β-hCG levels are significantly elevated in gestational trophoblastic disease (hydatidiform mole and choriocarcinoma among others). In cases of malignant germ cell tumors, some pure germ cell tumors (testicular seminoma, ovarian dysgerminoma, intracranial germ cell tumor), and other tumors such as liver tumors, pancreatic tumors, combined with elevated β-hCG indicate the presence of trophoblast cells (16). Two factors have been postulated to contribute to elevation of β-HCG in Gb patients. First, Gb secretes HCG. Ishida (5) hypothesized that histologically undifferentiated germ cells can secrete HCG. Additionally, HCG can be secreted by cells that are not germ cells. In 2022, Maeyama T (17) reported a case of HCG-secreting neuroblastoma (right adrenal region) causing peripheral precocious puberty in a 2-year-old boy. Immunohistochemical analysis of tumor tissues using serial sections showed that β-hCG was present in synaptophysin-positive cells, while immunofluorescence showed β-hCG co-stained with neuron-specific enolase. Second, Gb does not secrete HCG, but, there may be sporadic trophoblast cells in its tissue that can secrete HCG, which require a highly experienced pathologist to observe on the film. In the same way that pure seminoma does not secrete HCG, trophoblast cells can be seen in tumor tissues of seminoma patients with elevated HCG levels (18). Serum HCG levels are elevated in patients with Gb and dysgerminoma. Histopathological examination of the tumor reveals syncytiotrophoblast giant cells scattered in cells/stromal cells resembling primordial germ cells and immature Sertoli cells (19).

Clinical incidences of Gb-secreting HCG have not been conclusively reported. We agree with Schanne (8) that, in patients with a known risk of Gb, Gb results in elevated β-HCG levels and therefore, there is no need for searching for other causes of elevated β-HCG. In this case, due to elevated peripheral blood β-hCG levels, imaging examinations for extragonadal lesions, including the cranial brain, were performed in two hospitals. The significance of elevated β-HCG levels in Gb patients and the role that it plays as a tumor marker should be elucidated.

Venous blood collection from endocrine glands is commonly used for auxiliary localization of lesions, and despite significant advances in imaging technologies in recent decades, it continues to have a significant diagnostic significance. In a previous report of Gb with secreted PRL (15, 20), a 46, XX girl had a left pelvic mass with peripheral precocious puberty, pathologically suggesting Gb. Intraoperative peripheral venous blood PRL levels were 11.6 ng/L (RIA, normal <12), while tumor venous blood PRL levels were 95.6 ng/L, suggesting that Gb secretes PRL but its significance was unknown. As mentioned earlier, the first report of Gb that secreted HCG (5) discovered that the levels of *E*_2_ and T were increased in the effluent of tumor tissue and peripheral blood. In addition, β-HCG levels were elevated, which supports the notion that Gb secretes *E*_2_, T, and β-HCG. In our case, due to appearance of the tumor on the left side of the gonads and the cord-like appearance on the right-side during surgery, the tumor was biochemically diagnosed as secreting *E*_2_, T, and HCG on both sides, and this diagnosis was pathologically confirmed. Testing for gonad venous blood hormone concentrations will inform the surgeon/endocrinologist on assessment of the tumor lesion to decide on whether or not surgical excision is necessary.

Generally, Gb is considered to be a benign tumor (malignant premalignant state), and successful surgical excision of the diseased gonad is associated with good prognostic outcomes. Morin j (21) reported that the 5-year overall survival (OS) and recurrence-free survival rates (RFS) for 2,037 patients who underwent gonadal surgery for gonadal dysgenesis were comparable and >96% compared with patients who did not have Gb or germ cell tumors (GCTs). Besides, the 5-year OS and RFS rates for GCT patients following surgery were significantly decreased to 82.4% and 86.7%, respectively. However, in Gb or GCTs secreting HCG, the prognosis is unknown. None of the 4 reported cases had long-term follow-up.

This patient's pathology was Gb, a premalignant condition that was surgically removed and was in stage I of a childhood ovarian GCT stage (22). Due to elevated β-HCG levels, the patient received 4 courses of JEB chemotherapy. After surgical intervention, β-HCG levels were consistently below the detection limit. At the same time, growth retardation for this TS patient was present, preoperative BA was significantly advanced, growth potential had significantly diminished, and there was a strong need for GH application. However, the patient had elevated β-HCG levels, and it is possible that the tumor tissue contained components of trophoblasts that have yet to be identified. It is important to exercise extreme caution when administering GH to this patient. GH and IGF1 have mitogenic and pro-proliferative properties, and because of this, GH is contraindicated in patients with active tumors (22, 23). Although the currently published data on childhood and adult cancer survivors do not suggest that GH replacement increases future cancer risk, GH therapy, either by itself or in interaction with radiation and/or chemotherapy, is controversial with regards to the risk of tumor recurrence or occurrence of a second tumor (23, 24). However, there is a lack of consensus on clinical uses of GH for cancer survivors. Therefore, before administering growth hormones to this patient, it is vital to comprehensively assess the condition of the child and maintain effective communication with parents. After a year of the condition remaining stable with no evidence of recurrence, we concluded that beginning GH treatment while closely monitoring the patient was the best course of action.

In conclusion, we present the fifth case of HCG-secreting Gb in the world. It is a rare case of 45, X TS with gonad Y chromosome mosaicism. The patient presented with breast enlargement and cystic mammary glands, with virilizing features (clitoral hypertrophy, elevated T), and elevated blood β-HCG levels. Even though the patient was a female child, she exhibited masculine characteristics, thus, detection of peripheral blood karyotype was very important. When a TS patient exhibits masculinizing characteristics, it is necessary to investigate Y chromosome material to determine whether there is cellular mosaicism. Additionally, gonadal and adrenal tumors should be explored. Detection of Y chromosomal material in pathological tissues is important for diagnosis of gonadal Y chromosome mosaicism. In diagnosis and treatment of precocious puberty, clinicians should follow the routine of precocious puberty, conduct a comprehensive physical examination, carefully evaluate the examination report, detect abnormalities in a timely manner, and make early diagnosis as well as treatment. Patients with precocious puberty, regardless of gender, must undergo HCG and AFP examination.

## Data Availability

The original contributions presented in the study are included in the article/Supplementary Material, further inquiries can be directed to the corresponding author/s.
